# Isolation, Culture, and Differentiation of Bovine Muscle Resident Stem Cells

**DOI:** 10.21769/BioProtoc.5647

**Published:** 2026-04-05

**Authors:** Perri Gish, Madison W. Stewart, Maykal Tsonov, Brandon Khuu, Rachel Espinoza, Payam Vahmani, Lucas R. Smith

**Affiliations:** 1 Department of Animal Science, College of Agricultural and Environmental Sciences, University of California, Davis, CA, USA; 2 Department of Neurobiology, Physiology, & Behavior, College of Biological Sciences, University of California, Davis, CA, USA; 3 Department of Physical Medicine and Rehabilitation, School of Medicine, University of California, Davis, CA, USA

**Keywords:** Bovine muscle resident stem cells, Fibro-adipogenic progenitor cell, Muscle satellite cell, Primary cell isolation, Adipogenic differentiation

## Abstract

Bovine muscle satellite cells (MuSC) and fibro-adipogenic progenitor cells (FAP) are muscle resident stem cells that are responsible for postnatal muscle growth, intramuscular fat deposition, and extracellular matrix generation. These cells are of increasing interest for the cultivated meat community due to their ability to generate all the major components of meat; additionally, these cells are of interest to conventional animal science research to elucidate mechanisms to improve meat quality. To use these cells for these goals, efficient and accurate cell isolation, culture, and differentiation are essential to evaluate their cell fate decisions and behaviors. In this protocol, we detail a simultaneous isolation of both MuSCs and FAPs with multiple intermediate stopping points, allowing for flexibility for day-of time constraints. We also detail improved growth conditions to maximize cell expansion and procedures to assess cell differentiation. This protocol provides a flexible isolation procedure that is compatible with sampling in modern slaughterhouses or from biopsies. Additionally, the differentiation procedures provide improved differentiation but still allow in vitro treatment and assessment.

Key features

• This protocol offers a flexible in-lab procedure to isolate bovine FAPs and MuSCs from tissue collected post-slaughter with multiple pause points.

• The protocol demonstrates successful conditions to grow, expand, and differentiate bovine FAPs with an optimized adipogenic differentiation medium.

• Strategies for planning your primary cell isolation, choosing the sampling location, and characterizing differentiation of bovine FAPs and MuSCs are included.

## Graphical overview


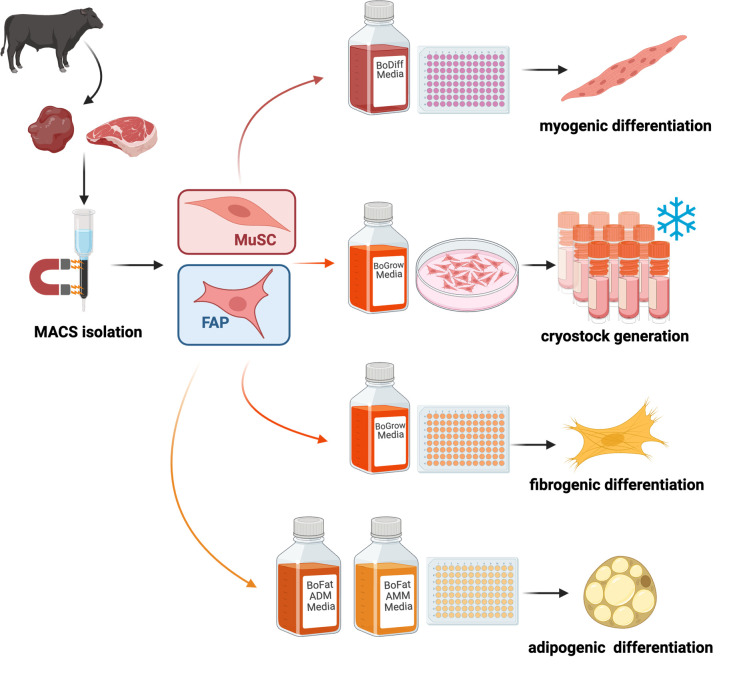


**Isolation and differentiation of bovine muscle resident stem cells.** MuSC, muscle satellite cells; FAP, fibro-adipogenic progenitor cells.

## Background

Skeletal muscle tissue is the predominant form of tissue consumed as meat [1]. Critical muscle structures for meat include contractile myofibers, which provide the majority of protein, lipids stored as fat, which provide flavor, and the extracellular matrix, which is integral to texture [2–6]. The development and growth of skeletal muscle rely on stem cell differentiation into these muscle structures. Each of these structures arises from muscle resident stem cells, with muscle stem cells (MuSC) or satellite cells as a progenitor to myofibers and fibro-adipogenic progenitors (FAP) which are capable of adipogenic differentiation into adipocytes or activation into prolific extracellular matrix-producing myofibroblasts [7,8]. Thus, knowledge of how these stem cells respond to a variety of stimuli is essential to understanding the adaptations of muscle tissue and thus meat quality [9,10]. The isolation of these stem cells provides the opportunity to explicitly control their environment or alter the cells using the plethora of cell biology tools available [10,11]. While many of these tools have focused on biomedical applications, isolation and culture of MuSCs and FAPs from agriculturally relevant species is vital to meat science [12,13]. Particularly, in the emerging field of cultivated meat, which utilizes in vitro methods to create meat, investigation of MuSCs and FAPs is essential [14,15].

Beef is the most studied meat, with many factors such as marbling impacting meat quality [16]. Thus, bovine MuSCs and FAPs are among the most commonly studied in meat science. Although these cells are the focus of this protocol, it may also serve as a template for other meats such as pork, poultry, or fish. Isolation of bovine MuSCs has been performed extensively, using a variety of protocols, while FAP isolation is increasingly being performed [17–21]. Pre-plating techniques that rely on differential adhesion dynamics of stem cell types allow enrichment of MuSCs in a simple, low-resource, intensive manner [22]. Fluorescence-activated cell sorting (FACS) alternatively provides high-purity stem cell populations but is resource-intensive and stressful to primary cells [23]. Magnetic activated cell sorting (MACS) offers a compromise to more selectively sort cells than pre-plating techniques while also being more accessible and gentler to cells than FACS [24]. Following isolation, these adherent cell types must be expanded in vitro. Many factors of the in vitro environment, from media composition to adhesion substrate, can impact cell behavior. The differentiation of stem cells is a key behavior, and a number of different media are used for proliferation or myogenic differentiation of MuSCs or adipogenic differentiation of FAPs [25–28]. Here, we provide a protocol for the simultaneous isolation and in vitro investigation of bovine MuSCs and FAPs. These protocols include coating procedures and multi-stage media compositions for differentiation that have been optimized for bovine stem cells [18]. These protocols establish a reproducible framework for in vitro studies of bovine stem cell contributions to meat development and quality.

## Materials and reagents

**Biological materials**

1. Bovine primary fibro-adipogenic progenitor cells (generated as part of this protocol)

2. Bovine primary muscle satellite cells (generated as part of this protocol)

**Reagents**

1. F10 media (Fisher Scientific, catalog number: 11550043)

2. Debris removal solution (Miltenyi Biotec, catalog number: 130-109-398)

3. CD140a (PDGFRα) Microbead kit, mouse (Miltenyi Biotec, catalog number: 130-101-547)

4. Satellite Cell Isolation kit, mouse (Miltenyi Biotec, catalog number: 130-104-268)

5. 0.25% Trypsin-EDTA (Fisher Scientific, catalog number: 25-200-056)

6. Collagenase type II powder (Fisher Scientific, catalog number: 17101015)

7. EDTA (Affymetrix, catalog number: 15694)

8. EDTA salt (Fisher Scientific, catalog number: BP120 500)

9. Bovine serum albumin (BSA) (Fisher Scientific, catalog number: 9048-46-8)

10. Penicillin-Streptomycin (10,000 U/mL) (Thermofisher Scientific, catalog number: 15140122)

11. Amphotericin B (Thermofisher Scientific, catalog number: 15290018)

12. Gentamicin solution (Thermofisher Scientific, catalog number: 15710064)

13. DMEM, high glucose (Thermofisher Scientific, catalog number: 11-965-092)

14. Ammonium chloride salt (Thermofisher Scientific, catalog number: 012361-36)

15. Sodium bicarbonate (Thermofisher Scientific, catalog number: 50-287-33)

16. Fibronectin solution (Advanced Biomatrix, catalog number: 5050)

17. PBS pH 7.2 (Thermofisher Scientific, catalog number: 20012027)

18. FBS (Biowest, catalog number: S1620)

19. FGF2 (ThermoFisher, catalog number: PHG6015)

20. DMSO (Thermofisher Scientific, catalog number: BP231-100)

21. GlutaMAX (Thermofisher Scientific, catalog number: 35050061)

22. Troglitazone (Thermofisher Scientific, catalog number: 501150786)

23. Insulin, human recombinant (Millipore Sigma, catalog number: 91077C-100MG)

24. IBMX (VWR, catalog number: 102516-252)

25. Dexamethasone (Thermofisher Scientific, catalog number: 1126100)

26. Trypan blue (Thermofisher Scientific, catalog number: C10228)

27. Paraformaldehyde (PFA), 16% w/v aq. soln., methanol free (Thermofisher Scientific, catalog number: 043368.9M)

28. Perilipin-1 (D1D8) rabbit monoclonal antibody (Cell Signaling, catalog number: 9349)

29. Actin, smooth muscle, Ab-1 mouse monoclonal antibody, Epredia^TM^ (Fisher Scientific, catalog number: MS113P1)

30. Hoechst 33342, trihydrochloride trihydrate (Invitrogen, catalog number: H3570)

31. Acti-stain 555 phalloidin (Cytoskeleton, Inc., catalog number: PHDH1)

32. Desmin antibody (B-7) (Santa Cruz Biotechnology, catalog number: sc-271677)

33. Myogenin polyclonal antibody (Invitrogen, catalog number: PA5-116750)

34. Donkey anti-rabbit IgG (H+L) highly cross-adsorbed secondary antibody, Alexa Fluor^TM^ Plus 647 (Invitrogen, catalog number: A32795)

35. Donkey anti-mouse IgG (H+L) highly cross-adsorbed secondary antibody, Alexa Fluor^TM^ Plus 488 (Invitrogen, catalog number: A32766)

36. Nile Red (ThermoFisher, catalog number: N1142)

37. Oil Red O (ThermoFisher, catalog number: A12989.22)

38. 70% v/v denatured ethanol (ThermoFisher, catalog number: BP82031GAL)

39. Triton X-100 (Fisher Scientific, catalog number: BP151-100)

**Solutions**

1. PEB (see Recipes)

2. Collagenase (see Recipes)

3. PBS antibiotic (see Recipes)

4. Erythrocyte lysis buffer (ACK) 10× (see Recipes)

5. Fibronectin coating solution (see Recipes)

6. BoFreeze media (see Recipes)

7. BoGrow media (see Recipes)

8. BoFat adipogenic differentiation media (ADM) (see Recipes)

9. BoFat adipogenic maturation media (MM) (see Recipes)

10. BoDiff media (see Recipes)

**Recipes**

**1. PEB**

**Table:** 

Reagent	Stock concentration	Final concentration	Quantity or volume
BSA	n/a	5 g/L	0.2 g
PBS	1×	n/a	40 mL
EDTA	0.5 M	2 mM	160 mL
Total	n/a	n/a	40 mL

Weigh out BSA and transfer to a sterile 50 mL conical tube. On the day of use, in a biosafety cabinet, add the EDTA and PBS and vortex to combine.

*Note: Some foam will form. You may allow 1–3 h at 2–8 °C for it to settle or aspirate the foam and use the liquid immediately. The BSA must be solubilized on the day of use, and the solution must always be made fresh.*

Store at 2–8 °C for up to 16 h and let it rise to room temperature before use.

**2. Collagenase**

**Table:** 

Reagent	Stock concentration	Final concentration	Quantity or volume
Collagenase	Variable, >125 U/mg	10,000 units	Variable
DMEM	n/a	n/a	5 mL
Pen/Strep	10,000 U/mL	2%	100 mL
Total	n/a	n/a	5 mL

Weigh out collagenase and transfer it to a sterile 15 mL conical tube. Adjust collagenase weight based on your reagent’s units/g specification.

Bring this into a biosafety cabinet and add pen/strep and DMEM. Homogenize via vortex or pipetting up and down until all solids are dissolved. Once dissolved, centrifuge at 300× *g* for 5 min. If solids remain after centrifugation, repeat homogenization and centrifugation as needed. Store at 2–8 °C for up to 2 weeks, and heat using a 37 °C water bath before use.

*Note: This recipe makes 5 mL of solution, which is sufficient for 10 g of tissue (0.5 mL/1 g). Scale up or down as needed.*

**3. PBS antibiotic**

**Table:** 

Reagent	Stock concentration	Final concentration	Quantity or volume
PBS	1×	n/a	49 mL
Pen/Strep	10,000 U/mL	1%	500 mL
Gentamicin	10 mg/mL	0.5%	250 mL
Amphotericin B	250 μg/mL	0.4%	200 mL
Total	n/a	n/a	50 mL

Add from largest to smallest volume using sterile technique in a biosafety cabinet. Store at 2–8 °C for up to 3 months.

**4. ACK (10**×)

**Table:** 

Reagent	Stock concentration	Final concentration	Quantity or volume
DMEM	n/a	n/a	~50 mL
Ammonium chloride	n/a	80.2 g/L	4.01 g
Sodium bicarbonate	n/a	8.4 g/L	0.42 g
EDTA salt	n/a	3.7 g/L	0.185 g
Total	n/a	n/a	50 mL

Weigh out all solids and transfer them into a 50 mL conical tube. Bring this into a biosafety cabinet and add DMEM up to the 50 mL line. Use a vortex or pipette up and down to mix.

*Note: Solids are resistant to dissolution and may require time overnight to fully dissolve. Additionally, bubbles will form once DMEM is added due to the bicarbonate.*

Filter-sterilize through a 0.22 mm filter and store at 2–8 °C for up to 3 months. Once ready to use, dilute this solution 1:9 with PBS to create a 1× solution.

**5. Fibronectin coating solution**

**Table:** 

Reagent	Stock concentration	Final concentration	Quantity or volume
PBS	1×	n/a	1 mL
Fibronectin	0.5 mg/mL	5 mg/mL	10 mL
Total	n/a	n/a	1.01 mL

Thaw a fibronectin aliquot at room temperature in a biosafety cabinet. Once thawed, combine the described volume with PBS, flushing out the pipette tip by pipetting up and down 2–3× when adding the fibronectin into your working solution. Vortex to homogenize and use immediately. To coat, in a biosafety cabinet, apply the working solution to your culture vessel to completely cover the vessel bottom and incubate at room temperature for 1 h; see Table 1 for suggested coating volumes. Aspirate the working solution, with care not to touch the bottom of the culture vessel, and wash 2–3 times with 1× PBS. Alternatively, the remaining solution may be collected, stored at -20 °C for 3 months, and reused up to 3×. Coated plates should be covered with ample amounts of 1× PBS, sealed with parafilm, and stored at 2–8 °C for up to 3 months. Discard plates if they dry out. Scale up the recipe as needed to thoroughly cover the bottom of your culturing vessel. This achieves a 1.56 mg/cm^2^ coating density.

**Table 1.** Recommended seeding densities and media volumes for culture

Vessel	Growth area (cm^2^)	Cells to seed for growth	Wash, trypsin, or coating solution volume (mL)	Media volume (mL)
**96-well plate**	0.32/well	1,650	0.05/well	0.100/well
**6-well plate**	9.6/well	40,000	0.5/well	2/well
**T25**	25	125,000	1	5
**T75**	75	375,000	3	15
**T182**	182	900,000	5	25

**6. BoFreeze media**

**Table:** 

Reagent	Stock concentration	Final concentration	Quantity or volume
FBS	n/a	90%	9 mL
DMSO	>99.7%	10%	1 mL
Total	n/a	n/a	10 mL

Add the FBS and then DMSO into a sterile 15 mL conical tube in a biosafety cabinet; invert 3× to homogenize. Store at 2–8 °C for up to 3 months, and warm to room temperature before use.

**7. BoGrow media**

**Table:** 

Reagent	Stock concentration	Final concentration	Quantity or volume
F-10 media	n/a	n/a	39mL
FBS	n/a	20%	10 mL
Pen/Strep	10,000 U/mL	1%	500 mL
Glutamax	100×	1%	500 µL
Gentamicin	10 mg/mL	50 mg/mL	250 mL
Amphotericin B	250 μg/mL	1 μg/mL	200 µL
FGF2	2,500 ng/mL	5 ng/mL	100 mL
Total	n/a	n/a	50 mL

Combine all reagents from largest to smallest volume in a 50 mL conical tube in a biosafety cabinet. Invert three times to homogenize, and optionally filter-sterilize with a 0.22 mm filter. Store at 2–8 °C for up to 3 months, and warm in a 37 °C water bath prior to use. This recipe may be scaled up to make larger stocks of media, but aliquots no larger than 50 mL should be made to reduce temperature exposure of the whole stock.

**8. BoFat ADM**

**Table:** 

Reagent	Stock concentration	Final concentration	Quantity or volume
BoGrow	n/a	n/a	4.864 mL
Insulin	2 mg/mL	5.258 mM	76.34 mL
Troglitazone	2.5 mg/mL	5 mM	44.15 mL
Dexamethasone	10 mM	1 mM	5 mL
IBMX	250 mM	500 mM	10 mL
Total	n/a	n/a	5 mL

Thaw all adipogenic-inducing reagents in a 37 °C water bath and then transfer to a biosafety cabinet. Add reagents from largest to smallest volume in a sterile 5 or 15 mL conical tube and vortex or invert. Some precipitate may form, which will require time and heat to resolubilize. Use the media the next day or heat in a 37 °C water bath for ~30 min to encourage solubilization. Filter sterilization through a 0.22 mm filter may be optionally conducted after all precipitate is resolubilized. Store at 2–8 °C for up to 3 months.

**9. BoFat AMM**

**Table:** 

Reagent	Stock concentration	Final concentration	Quantity or volume
BoGrow	n/a	n/a	4.879 mL
Insulin	2 mg/mL	5.258 mM	76.34 mL
Troglitazone	2.5 mg/mL	5 mM	44.15 mL
Total	n/a	n/a	5 mL

Thaw all adipogenic-inducing reagents in a 37 °C water bath and then transfer to a biosafety cabinet. Add reagents from largest to smallest volume in a sterile 5 or 15 mL conical tube and vortex or invert. Some precipitate may form, which will require time and heat to resolubilize. Use the media the next day or heat in a 37 °C water bath for ~30 min to encourage solubilization. Filter sterilization through a 0.22 mm filter may be optionally conducted after all precipitate is resolubilized. Store at 2–8 °C for up to 3 months.

**10. BoDiff media**

**Table:** 

Reagent	Stock concentration	Final concentration	Quantity or volume
DMEM	n/a	n/a	9.7 mL
FBS	n/a	2%	200 µL
P/S	10,000 U/mL	1%	100 µL
Total	n/a	n/a	10 mL

Combine all reagents from largest to smallest volume in a 15 mL conical tube in a biosafety cabinet. Invert three times to homogenize, and optionally filter-sterilize with a 0.22 μm filter. This recipe may be scaled up to make larger stocks of media, but aliquots should be no larger than 50 mL. Store at 2–8 °C for up to 3 months, and warm in a 37 °C water bath prior to use.

**Laboratory supplies**

1. 15 mL conical centrifuge tubes, polypropylene, sterile (Genesee, catalog number: 28-101)

2. 50 mL conical centrifuge tubes (Genesee, catalog number: 28-106)

3. Olympus Premium 0.6 mL snap cap microcentrifuge tubes (Genesee, catalog number: 24-272)

4. Petri dish 150 mm × 22 mm (Genesee, catalog number: 32-106)

5. P10–P1000 tips, sterile (USA Scientific, catalog numbers: 1112-1720, 1110-1700, 1110-3700)

6. Serological pipettes, 5–50 mL, sterile (Genesee, catalog numbers: 12-102, 12-104, 12-106, 12-107)

7. TC plates of various sizes: 96 well, 6 well, T25, T75 (Genesee, catalog numbers: 25-109, 25-105, 25-207, 25-209)

8. MACS C Tube (Miltenyi, catalog number: 130-093-237)

## Equipment

1. Biological Safety Cabinet 1300 Clas II Type A2 (Fisher Scientific, catalog number: 1911310)

2. Water bath capable of reaching 37 °C

3. Freezer (-20 °C)

4. Refrigerator (2–8 °C)

5. Liquid nitrogen tank

6. Serological pipettes and micropipettes (standard sizes, sterile)

7. Incubator capable of maintaining 37 °C and 5% CO_2_

8. Microscope capable of 10×, 20×, and 40× magnification in brightfield

9. Hemocytometer

10. Countess (ThermoFisher) or other automated cell counter

11. Warming oven capable of reaching 37 °C

12. MACSmix tube rotator (Miltenyi Biotec, catalog number: 130-090-753)

13. Mini & MidiMACS Starting kit (Miltenyi Biotec, catalog number: 130-042-501)

14. gentleMACS dissociator (Miltenyi Biotec, catalog number: 130-093-235)

## Software and datasets

1. FIJI (ImageJ2, version 2.16.0/1.54p); FIJI is ImageJ with many plugins already added. This is a free-to-use, open-source software that can be downloaded at www.imagej.net/software/fiji/downloads

## Procedure

**A. Isolation of FAPs and MuSCs**

**A1. Dissection and tissue dissociation (~30 min)**

1. Prepare the following ahead of the day of sampling: fibronectin-coated plates, BoGrow, and collagenase solution. We also recommend prelabeling all tubes used for isolation to improve clarity and efficiency during the protocol. Further details for prelabeling tubes, and a reference to the steps in which they are used, can be found in Table 2.

**Table 2.** Tubes needed for isolation, with label and associated step in the protocol

Label	Tube size (mL)	Section used in	Steps used in
A	50	A2	1–6
B	50	A2	2–7
C	50	A2	8–10
D	15	A2	10–17
E	50	A2	16–19
F	50	A2	19–20
G	15	A2	20–22
H	15	A3	1–8
Unlabeled cells	15	A3	5–16
FAPs	15	A3	9–14
Wash	15	A3	11–15
MuSCs	15	A3	15–17

2. Obtain a fresh (≤30 min after slaughter/biopsy/excision) piece of bovine skeletal muscle. Up to 4 g may be used before needing to scale up reagents and consumables. Immediately place the sample into a 50 mL conical tube pre-filled with 20 mL of PBS antibiotic solution (see Recipes), a sufficient volume to completely cover the tissue. Transport the tissue in the 50 mL tube on ice to the laboratory.

*Notes:*

*1. For sampling from commercially slaughtered cattle, we recommend collecting muscle from the brisket to work seamlessly with conventional carcass preparation and to reduce time between slaughter and the isolation procedure.*

*2. Our tissue transport time averages 5 min. We recommend limiting the time between sample collection and beginning step A1.3 to ~30 min to preserve cell viability. The procedure may be attempted with longer times, but cell yields are expected to decrease as time increases.*

3. In a biosafety cabinet, fill one 150 mm × 22 mm Petri dish with 70% ethanol and fill another three dishes with PBS.

*Note: Both the bottoms and lids of the Petri dish may be used; no cover is needed. Alternatively, a 50 mL conical tube may be used.*

4. Transfer the tissue using sterile forceps from the 50 mL conical tube into the 70% ethanol–filled dish and set a timer for 5 min to disinfect. After 5 min, transfer the tissue into the first PBS dish to wash for 3–5 min; repeat for all three PBS dishes. During the 5-min disinfection period, place the DMEM and collagenase solution into a 37 °C water bath to warm.

5. In the last PBS dish after the 3–5 min disinfection period, dissect the muscle using sterile forceps and scissors, taking care to remove any large pieces of intermuscular fat or connective tissue; marbling fat may be left in.

6. Weigh the minced muscle to a 1–4 g sample and transfer it to a MACS C tube.

*Note: Larger tissue samples will have more debris and increase isolation time during the filtering steps. For regular isolations, we recommend using ~2–3 g of tissue to digest.*

7. In the biosafety cabinet, add 5 mL of DMEM to the MACS C tube with the muscle sample. Then, add 0.5 mL of collagenase solution per 1 g of tissue to digest, e.g., for 2.25 g of tissue, you will add 1.13 mL of collagenase.

8. Seal the MACS C tube and affix it into a MACS tube rotator inside a warming oven set to 37 °C. Set the rotator to continuously spin and set a timer for 30 min.

9. After 30 min, retrieve the MACS C tube and place it into the gentleMACS dissociator. Run the manufacturer-provided program “m_muscle_01” and then return the tube to the rotator in the warming oven.

10. After 30 min, repeat steps A1.8–9 for a total of two blending steps.

**A2. Cleaning (~1.5 h)**

**Figure 1.**
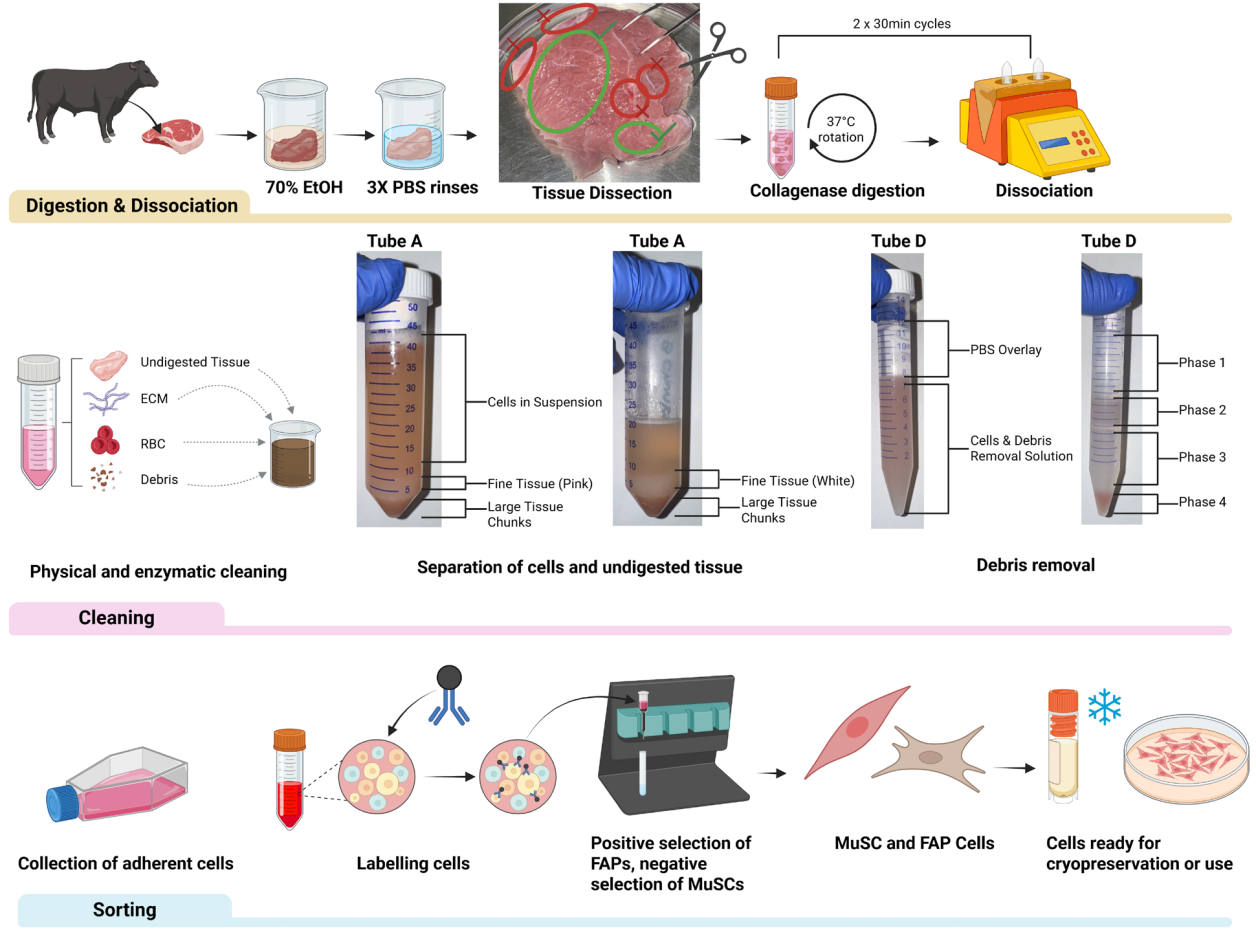
Isolation of bovine fibro-adipogenic progenitor cells (FAPs) and muscle satellite cells (MuSCs). The image depicts the three phases of the isolation process: digestion and dissociation, cleaning, and sorting. An image of veal skeletal muscle with areas to use for isolation (green circles) versus areas not to use (red circles) are shown. Example images of the separation of cells from undigested tissue and of the debris removal process are shown in the cleaning phase.

1. After the second dissociation, bring the MACS C tube into the biosafety cabinet. Transfer all MACS C tube contents into a 50 mL conical tube labeled “A.” Rinse the MACS C tube twice with 5 mL of PBS and a vigorous shake to dislodge any stuck tissue. Add these rinses to the MACS C tube. Centrifuge tube A at 70× *g* for 3 min at 4 °C.

2. Transfer the supernatant to a new 50 mL tube labeled “B” and centrifuge at 500× *g* for 5 min at 4 °C.

3. Add 20 mL of PBS to A and invert 2–3 times. Take note of the color of the sediment on top of any undigested tissue in tube A; refer to Figure 1 for a reference.

4. After tube B is finished spinning, place tube A in the centrifuge for another 3 min at 70× *g* at 4 °C.

5. During spinning, aspirate the supernatant in tube B and resuspend the pellet in 25 mL of PBS. Set this aside.

6. If space allows, collect the supernatant from tube A into tube B, and then into another 50 mL conical tube if needed. Repeat the washing of the undigested tissue, centrifugation, and collection of the supernatant in tube A until the sediment appears white in color. Refer to Figure 1 for a visual reference; this typically takes ~3 repetitions. Once the sediment is white, tube A may be discarded.

7. Centrifuge all supernatant-containing tubes, including tube B, at 500× *g* for 5 min at 4 °C. Resuspend all pellets in 10 mL of PBS.

8. Pass all resuspended pellets through a 100 μm filter affixed to a new 50 mL conical tube labeled “C.” All pellets should be collected.

*Note: If the suspension is resistant to passing through the filter, gently pipette up and down on the suspension using a P1000 tip. You may also gently stir the suspension on the filter using the bottom side of a P10 tip, taking care not to puncture the filter. The filtering is sufficient when there is <500 µL remaining.*

9. Centrifuge tube C at 300× *g* for 10 min at 4 °C, aspirating the resulting supernatant and resuspending the cell pellet in 2.2 mL of cold PBS.

10. Move the cell suspension to a 15 mL conical tube labeled “D.” Rinse tube C two times with 2 mL of cold PBS, adding the rinse to tube D. The volume in the 15 mL tube should now be ~6.2 mL and cold to the touch.

11. Add 1.8 mL of cold debris removal solution to tube D. Using a 5 mL serological pipette, mix by pipetting up and down 10 times.

12. Tilt the tube D slightly to a ~30 ° angle. Using a P1000, slowly overlay with 4 mL of cold PBS. Do not mix phases; refer to Figure 1 for reference to a successful overlay.

13. Centrifuge tube D at 3,000× *g* for 10 min at 4 °C. During this time, warm the ACK, F10 media, PBS aliquot, and BoGrow in a 37 °C water bath.

14. Aspirate and discard the top two phases. Once approaching the second, cloudy phase, gently puncture it with your aspirating pipette to remove it without it dipping too far into the third phase. Refer to Figure 1 for a phase reference.

15. Add cold PBS to the 15 mL line and invert tube D three times. Then, centrifuge tube D at 1,000× *g* for 10 min at 4 °C.

16. Aspirate and discard the supernatant and resuspend the pellet in 1 mL of PBS. Transfer to a new 50 mL conical tube labeled “E.”

17. In tube D from step A2.15, combine 1.5 mL of the ACK solution with 13.5 mL of PBS. Transfer this to tube E with the cell suspension and let it stand at room temperature for 1 min.

18. After 1 min, add PBS to the 50 mL line and centrifuge tube E at 500× *g* for 5 min at 4 °C. Aspirate the supernatant and resuspend in 30 mL of a 1:1 solution of F10 media and PBS.

19. Pass the suspension through a 40 µm filter affixed to a new 50 mL conical tube labeled “F.” Rinse tube E twice with 5 mL of PBS and pass through the filter into tube F as well.

20. Centrifuge tube F at 500× *g* for 5 min at 4 °C, resuspend in 2 mL of PBS, and transfer to a 15 mL conical tube labeled “G.” Rinse tube F three times with 3 mL of PBS and collect in tube G alongside the cells.

21. Centrifuge tube G at 300× *g* for 5 min at 4 °C. Resuspend the pellet in 1 mL of BoGrow.

22. Optionally, count cells. If counting, a hemocytometer is recommended as opposed to automated machines, e.g., Countess, to enhance accuracy with remaining debris.

**Pause point**: You may proceed immediately to cell sorting or plate the cells for 2–3 days. If plating cells, please note the following details:

1. Pausing for 3 days enriches for adherent cells (MuSCs and FAPs), allows cells time to recover, and allows for more isolations to be completed in one day.

2. Plate cells on a fibronectin-coated plate with BoGrow and let them rest in an incubator set to 37 °C and 5% CO_2_. For 2–4 g of tissue, a T25 is recommended. Do not aspirate the media during this time to avoid losing unadhered cells.

3. After 2–3 days (3 is preferred), collect the supernatant on the plates and freeze using the same cell freezing protocol described in section B1. In the event of unsuccessful isolation, you can thaw the supernatant to try sorting; this has worked previously in our lab, albeit at a low success rate. Otherwise, trypsinize and pull the cells as normally done during passaging and described in section B1 before proceeding with sorting.

**A3. Sorting (~2 h)**

1. Add PBS to the 5 mL line of a 15 mL conical tube labeled “H” that contains your cell suspension. Centrifuge at 300× *g* for 10 min at 4 °C.

*Notes:*

*1. This 15 mL tube is the former tube G from phase 2 if you did not use the pause point. We have relabeled it to “H” for this phase of the protocol for clarity for those who used the pause point.*

*2. Locate the Miltenyi CD140a (PDGFRα) Microbead kit during this time, and ensure PEB is made (see Recipes). Both the included FCR blocking reagent and the PDGFRα microbeads are used in the subsequent steps.*

2. Aspirate the supernatant and resuspend the cell pellet in 80 µL of fresh PEB. Add 10 µL of FCR blocking reagent and mix well by gently pipetting up and down while spinning your pipette tip slowly in a clockwise motion. Incubate tube H for 10 min in a 2–8 °C fridge.

3. Return tube H to a biosafety cabinet and add 10 µL of PDGFRα microbeads. Mix well as described in step A3.2. Incubate in a 2–8 °C fridge for 15 min.

4. After the incubation, add 2 mL of PEB to tube H and centrifuge at 300× *g* for 10 min at 4 °C.

5. Meanwhile, bring the MACS separator, mini/midi column holders, and an LS and MS column into the biosafety cabinet. Set up the mini (green) holder and an MS column on the separator with a fresh 15 mL tube labeled “unlabeled cells” underneath.

6. Add 500 mL of PEB directly to the column to rinse it, collecting the flowthrough in the “unlabeled cells” tube.

7. Retrieve tube H from the centrifuge, aspirate the supernatant, and resuspend the pellet in 500 mL of PEB. Apply this to the MS column and allow it to pass through.

*Note: Liquid should only be added to the column once the top of the column is free from liquid.*

8. In the now-empty tube H, add 1,800 mL of PEB. Wash the MS column with three rinses of 500 mL of PEB, using the PEB from the cell tube. All flowthrough should be collected in the “unlabeled” tube.

9. Once the column is dry and no liquid is dripping from the bottom, remove the MS column and place it into a new 15 mL conical tube labeled “FAPs.” Add 1 mL of PEB onto the column and immediately flush out the FAPs by firmly pushing the plunger into the column.

10. Centrifuge both the unlabeled cells and the FAPs at 300× *g* for 10 min at 4 °C.

11. Meanwhile, warm BoGrow media (see Recipes) in a 37 °C water bath and set up an LD column on the purple column holder. Set a tube labeled “wash” to collect the waste wash underneath. Pipette 3 mL of PEB onto the column and allow it to rinse the column.

12. Retrieve the two tubes from the centrifuge. Aspirate the supernatant on both and resuspend the unlabeled cells in 80 mL of PEB and the FAPs in 1 mL of warmed BoGrow media.

13. With the unlabeled cells, add 20 mL of the Satellite Cell Isolation kit. Mix well as described in step A3.2. Incubate in a 2–8 °C fridge for 15 min.

14. Meanwhile, count the FAPs using a hemocytometer. Then, plate FAPs on a fibronectin-coated plate in BoGrow media, aiming for a seeding density of ~2,000 cells/cm^2^. Store this plate in your cell culture incubator and culture as needed.

a. Typically, 1–2 wells of a 6-well plate are sufficient. A typical count for 2 g of starting tissue is ~50,000–100,000 live cells/mL.

b. If desired, you may freeze these cells immediately. However, this will reduce viability, and at least one passage is recommended before freezing.

15. After 15 min, retrieve the unlabeled cell tube from the fridge and add 420 mL of PEB. If the wash on the LD column is finished, you may discard the wash tube and replace it with a new 15 mL conical tube labeled “MuSCs.”

16. Apply the unlabeled cell suspension to the LD column, collecting the flowthrough in the MuSC tube. Once this has passed through, wash the LD column three times with 1 mL of PEB each rinse; collect this flowthrough in the same MuSC tube. On the last rinse, with the column in the holder affixed to the separator, use the plunger to push out the last rinse.

17. Centrifuge the MuSCs at 300× *g* for 5 min at 4 °C. Then, count and plate the MuSCs as described for FAPs in step A3.14. Typically, 2–3 wells of a 6-well plate are sufficient. An average yield from 2 g of tissue is ~80,000–200,000 live cells/mL.

**B. General culture and analysis of MuSCs and FAPs**

**B1. General cell culture**

1. Cells should be grown in a 37 °C incubator with 5% CO_2_. Media should be replaced with warmed, fresh media in each growing well every 2–3 days unless otherwise specified. Sterile technique in a biosafety cabinet using sterile reagents should *always* be used.

2. To maintain cells in growth conditions for colony expansion and preservation, cells should be passaged *before* the most confluent area in a vessel is 90% confluent. See Table 1 for the recommended seeding densities in common culture vessels for growth conditions to maximize cell yield, as well as recommended media and wash volumes.

*Note: We recommend performing all cultures on fibronectin-coated vessels. See general note 5 for more details.*

3. Follow the instructions below for cell passaging when needed:

a. In a 37 °C water bath, warm your trypsin-EDTA and BoGrow media.

b. In the biosafety cabinet, aspirate the old media without touching the adhered cells at the bottom of the vessel.

c. Add enough 1× PBS to the vessel to cover the entire bottom; for a T25 flask, 2 mL is sufficient. Gently rotate the flask to circulate the PBS over the entire adherent surface and then aspirate the PBS. Repeat this washing step twice to remove all trace amounts of cell media.

d. Add the same volume of 0.25% trypsin-EDTA as that of the PBS used in the washing step, ensuring the entire adherent surface is saturated. Return the vessel to the incubator and incubate for 5 min, allowing cells to detach.

*Note: Time is critical in this step. Do not let cells incubate too long with trypsin; otherwise, they may be digested by the reagent.*

e. After 5 min, confirm that the cells are sufficiently detached by gently hitting the side of the vessel and observing under the microscope. Detached cells should appear as floating circles, and adherent cells should no longer be visible. If cells are not sufficiently detached, incubate for another 2 min and check again.

f. Without removing the trypsin from the vessel, add BoGrow to the vessel; use 2–3 times the volume of trypsin used. Rotate gently for flasks or pipette up and down in plates to mix and halt the trypsin reaction.

*Note: An extra 2–5 mL of BoGrow media may be added to the empty flask to wash it, and the liquid can be added to the same conical tube.*

g. Transfer all the trypsin–BoGrow solution from your culture vessel to a 15 mL conical centrifuge tube. Centrifuge at 300× *g* for 5 min at 4 °C.

h. Retrieve your 15 mL tube and observe the cell pellet. Aspirate as much supernatant as possible without disturbing this pellet. Resuspend cells in 1 mL of BoGrow and gently pipette up and down to homogenize.

i. In a 0.6 mL Eppendorf tube, add 10 µL of trypan blue and 10 µL of your cell suspension. Gently pipette up and down 10 times to mix.

j. Using 10 µL of this trypan blue and cell mixture, count your cells and their viability and record the values. A Countess or hemocytometer may be used according to the instrument’s directions.

k. Using the live cell count, determine the volume of the cell suspension needed to seed your desired number of cells. Refer to Table 1 for the recommended seeding densities for growth conditions in common culture vessels.

l. To your new vessel, first add a sufficient volume of BoGrow, then add the calculated volume of your cell suspension. Gently shake the vessel twice in the vertical and then horizontal direction to evenly disperse cells across the adherent surface.

For FAPs and MuSCs, a vessel precoated with fibronectin is recommended. Please see the Recipes section for how to coat vessels. Fibronectin was found to improve FAP adhesion times and proliferation rates and can help maximize cell growth and yield [18].

m. Confirm via microscopy that cells have been successfully plated in your new vessel and return this vessel to your incubator to adhere and grow.

4. To freeze cells, follow steps B1.3a–j to detach and count your cells. Then, proceed with the following steps:

a. Warm BoFreeze media in a 37 °C water bath and locate cryotubes and a cool cell chamber.

b. Centrifuge the 1 mL cell suspension at 300× *g* for 5 min at 4 °C. During this time, calculate how many preserved vials you would like to make and prelabel your cryotubes with the cell line, passage number, current date, and number of live cells that each cryotube will contain. We recommend freezing cells at ≥1,000,000 live cells/cryotube.

c. Aspirate as much supernatant as possible without disturbing this pellet.

d. Resuspend cells in 1 mL of BoFreeze and gently pipette up and down to homogenize. Then, add this cell suspension to your cryotubes and seal. Once BoFreeze is added to cells, the following should be performed quickly, as DMSO can be toxic to cells at room temperature.

e. Place the sealed cryotubes into your cool cell chamber and set them inside a -80 °C freezer for 24 h. After 24 h, retrieve the cryotubes and store them in a cryotank in the vapor phase of liquid nitrogen.

f. To thaw cells, quickly warm the cryotube and twist the cap open to equalize pressure between the vial and ambient conditions. Then, warm the cell suspension until 90% thawed in a 37 °C water bath and proceed with counting and plating as detailed in steps B1.3g–m.

**B2. Cell fixation**

1. At the benchtop, carefully aspirate the media from each well, being sure not to disturb the bottom of the well.

2. Add 100 µL of 4% PFA to each well.

3. Incubate at room temperature for 10 min.

4. Wash the cells twice with PBS:

a. Remove the previous solution from the wells by gently aspirating.

b. Add 100 µL of PBS to each well.

c. Place the 96-well plate on a shaker table for 3 min.

5. Once two washes have been completed, add 200 µL of PBS to each well.

6. Wrap the edges of the plate with parafilm to prevent moisture loss. Store the assay plate at 2–5 °C for up to 3 months, making sure not to let the wells dry out, or proceed directly to step B3a.

**B3a. Staining day 1 (50–70 min)**

1. Prepare BSA solutions:

a. Prepare 10 mL of 5% BSA by adding 500 mg of stock BSA into 10 mL of PBS. Vortex until the BSA is completely dissolved.

b. Prepare 40 mL of 0.1% BSA by adding 800 mL of 5% BSA (see above) into 40 mL of PBS. Vortex to homogenize the solution.

*Note: BSA solutions can be stored at 2–4 °C for up to 1 week.*

2. Aspirate the PBS in plates/wells.

3. Add 60 µL of 0.5% Triton (v/v in 1× PBS) and shake for 10 min at room temperature.

4. Rinse each well twice with 100 µL of 0.1% BSA, shaking for 5 min between each rinse.

5. Add 100 µL of 5% BSA to each well and shake for 30 min.

a. Meanwhile, prepare 50 µL of primary antibody mix per well in 5% BSA.

*Note: All primary antibodies should be in one solution.*

6. After the 30 min have elapsed, add 50 µL of the antibody mixture to each well and then incubate the well plate on a shaker table at 4 °C overnight.

**B3b. Staining day 2 (140–160 min)**

1. Remove the well plate from 4 °C and rinse each well 3× with 100 μL of 0.1% BSA, shaking for 5 min between each rinse. Meanwhile, prepare 75 μL of secondary antibody mix per well in 0.1% BSA.

*Note: All secondary stains should be in one solution.*

2. Add antibody solution to each well and shake for 90 min in a dark box or wrapped in tin foil. All steps afterward must be conducted in a dark box.

3. Rinse each well three times with 100 µL of 0.1% BSA, shaking for 5 min between each rinse.

4. Add 75 μL of Hoechst (1:2,000 dilution) in 0.1% BSA per well.

5. Place on a shaker table for 15 min.

6. Rinse twice with 100 μL of 0.1% BSA and then once with PBS, *without* shaking.

7. Add 200 μL of PBS and store in a dark box at 2–4 °C for up to three months, sealing the edges of the plate with parafilm and making sure the wells do not dry out. Alternatively, proceed to imaging.

**C. Adipogenic and fibrogenic activation of FAPs**

**C1. Fibrogenic activation**

*Note: The following steps should be completed in a biosafety cabinet after cells have been counted according to section B1.*

1. Seed 50,000 FAP cells in 100 µL of BoGrow per well in triplicate in a 96-well plate.

a. Optional: Create a negative control well by seeding a fourth well with 50,000 cells in 100 µL of BoGrow in the opposite corner of the same 96-well plate or on another 96-well plate. Fix these cells according to section B2 after 24 h.

2. Incubate cells at 37 °C and 5% CO_2_ for 10 days.

*Notes:*

*1. BoGrow should be changed every 2–3 days during the assay.*

*2. Fibrogenic FAPs will grow in layers and are prone to peeling from the well surface under agitation. All media changes and the following steps should be done with hand aspiration using a P1000 and extreme care.*

3. After 10 days, proceed to fixation as detailed in section B2.

**C2. Adipogenic differentiation**

*Note: The following steps should be completed in a biosafety cabinet after cells have been counted according to section B1.*

1. Seed 50,000 FAP cells in 100 µL of BoGrow per well in triplicate in a 96-well plate.

a. Optional: Create a negative control well by seeding a fourth well with 50,000 cells in 100 µL of BoGrow in the opposite corner of the same 96-well plate or on another 96-well plate. Fix these cells according to section B2 after 24 h.

2. Incubate cells at 37 °C and 5% CO_2_ for 24 h.

3. Twenty-four hours post-seeding, carefully aspirate the BoGrow from each well, making sure not to touch the bottom of the wells.

4. Gently add 100 µL of BoFat ADM media to each of the wells. Return cells to the incubator and let them differentiate for 3 days.

*Note: Adipogenic FAPs become buoyant as they accumulate lipids. All media changes should be done using hand aspiration using a P1000, and extreme care should be taken to reduce detachment.*

5. After 3 days of differentiation, gently add 100 µL of BoFat AMM media to each of the wells. Return cells to the incubator and let them differentiate for 7 days.

**Figure 2.**
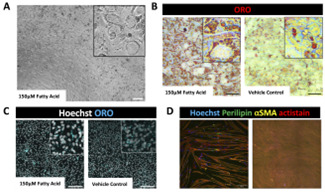
Differentiation of fibro-adipogenic progenitor cells (FAPs). (A) Brightfield image of day 3 FAPs in BoFat adipogenic differentiation media (ADM) with 150 μM fatty acid supplemented. (B) Brightfield image of fixed and stained adipogenically differentiated FAPs with and without fatty acid supplementation, showing lipids stained with Oil Red O (ORO). Scale bar, 50 μm. (C) Fluorescent images of adipogenically differentiated FAPs with and without fatty acid supplementation, showing lipids stained with ORO in blue and nuclei stained with Hoechst in white. Scale bar, 200 μm. (D) Fluorescent images of differentiated FAPs showing positive perilipin and aSMA expression in green and yellow, respectively. The second panel depicts fibrogenic FAPs growing in layers. Scale bars, 100 μm.

*Note: BoFAT should be changed every 2–3 days during maturation. Differentiation can be visually checked by looking at cell morphology. Differentiated cells should have visible lipid droplets and appear rounded. Refer to Figure 2.*

a. Should significant detachment be observed, the assay can be ended early. However, this should be noted and then standardized for all FAP assays in the experiment.

6. After 10 days of differentiation, add 50 µL of 16% PFA to the culture media in each well and follow the fixation steps detailed in section B2.

**C3. FAP staining and imaging**

1. FAP staining follows the general protocol detailed in section B3; however, all aspirations should be done by hand using a P1000, and all incubations with shaking should be done without shaking. The following primary and secondary stains should be used:

a. Primary stains: aSMA for fibrogenic FAPs (1:800 dilution) and perilipin-1 for lipids (1:200 dilution).

b. Secondary stains: donkey anti-mouse 647 antibody (1:500 dilution), donkey anti-rabbit 488 antibody (1:500 dilution), and acti-stain (1:250 dilution).

2. Alternatively, perilipin-1 stain and donkey anti-rabbit 488 may be replaced with Oil Red O (ORO) or Nile Red (NR) staining.

*Note: If ORO/NR is used, acti-stain must not be used, or an alternative phalloidin visible at 488 nm should be used*.

3. ORO/NR should be used after all immunofluorescent stains, using the following guidelines (always with protection from light) to make fresh working solutions:

a. For ORO, dilute ORO stock 3:2 with distilled water. Let this solution sit for 5 min and then filter through a 0.22 mm filter. ORO stock is 60 mg of dye in 20 mL of 100% isopropanol.

b. For NR, dilute NR stock 1:1,000 with 1× PBS. NR stock is 1 mg of dye with 3.14 mL of DMSO.

c. Apply 100 µL of working solution directly to the cells and set the plate in a dark box. Let this incubate for 15 min.

d. Aspirate the working solution and add 100 µL of 1× PBS to each well, let sit for 20 s, and then remove to wash. Repeat this 2–3 times or until the wash solution appears clear on the cells when using ORO.

e. Add 200 μL of PBS and store in a dark box at 2–4 °C for up to three months, sealing the edges of the plate with parafilm and making sure the wells do not dry out. Alternatively, proceed to imaging.

*Note: You can also read ORO/NR-stained plates in a plate reader to measure each well’s total absorbance of the stain. Excitation/emission maxima of NR/ORO are ~552/636 nm.*

4. Following staining, follow the steps below for imaging:

a. Hoechst stain for nuclei should be imaged at a wavelength of 461 nm.

b. aSMA should be imaged at a wavelength of 674 nm.

c. Perilipin or phalloidin should be imaged at a wavelength of 488 nm.

d. Acti-stain or ORO for actin should be imaged at a wavelength of 555 nm.

**C4. Image analysis**

**Figure 3.**
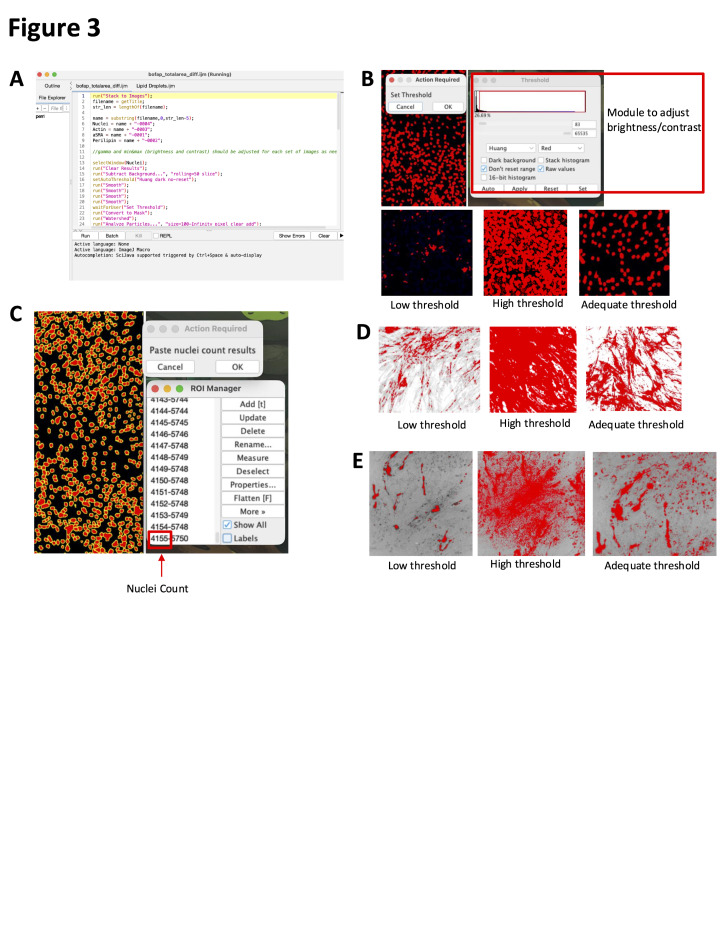
Fibro-adipogenic progenitor cell (FAP) differentiation area analysis. (A) bofap_totalarea_diff.ijm macro upon opening in FIJI. (B) Thresholding module and examples of thresholding on nuclei. (C) Output of nuclei counting; the bottom leftmost number shows the total nuclei count. (D) Thresholding examples for actin or aSMA. (E) Thresholding examples for lipids.

1. To measure the total lipid accumulating area and fibrogenic area, ensure you have the IJM macro “bofap_totalarea_diff.ijm,” your image files (.lif or .tiff), and the Excel “FAP_Diff_Analysis.xlsx” downloaded. The macro and Excel template can be found in the supplemental files, and Figure 3 depicts key items in this workflow. Then, follow the directions below:

a. Using Fiji2 (ImageJ), load the images of the differentiation wells and the macro entitled “bofap_totalarea_diff.ijm.”

b. In Microsoft Excel, open “FAP_Diff_Analysis.xlsx” and select the *Total Area* tab.

c. In Fiji, click *Run* in the macro panel.

d. When prompted, adjust the nuclei *threshold* such that all the nuclei are included, but any background fluorescence is not. Click *Ok* when finished.

e. When prompted, paste the nuclei results into the corresponding column of the Excel template. Click *Ok* when finished.

f. Repeat steps C4.1a–e for acti-stain, perilipin-1, and aSMA.

g. Use the calculated outputs in columns F–H for further analysis.

**Figure 4.**
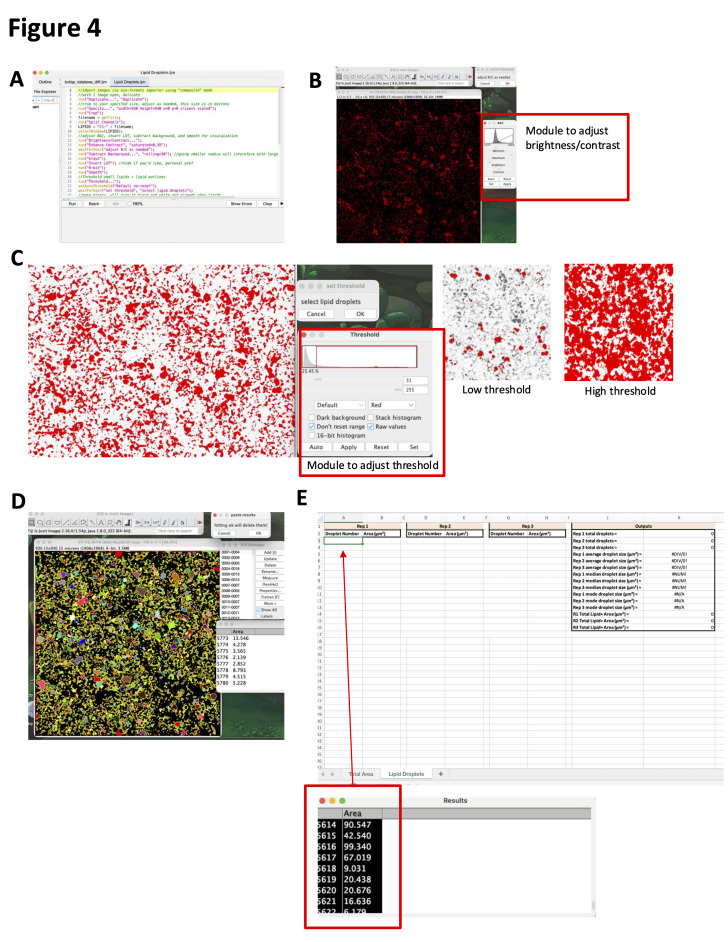
Lipid droplet analysis. (A) Lipid Droplets.ijm macro upon opening in FIJI. (B) Image of Oil Red O (ORO) lipid droplets after brightness/contrast adjustments, with the module to adjust shown. (C) Thresholding examples with the module to adjust are shown. (D) Masks and output windows for lipid droplet measurements. (E) Results of the .ijm macro are copied into a replicate’s column in the FAP_Diff_Analysis.xlsx.

2. To measure lipid droplet parameters, ensure you have the IJM macro “Lipid Droplets.ijm,” your image files (.lif or .tiff), and the Excel “FAP_Diff_Analysis.xlsx” downloaded. The macro and Excel template can be found in the supplemental files; please see Figure 4 for depictions of this analysis pipeline. Then, follow the directions below:

*Note: Lipid droplet analysis is optimized for ORO/NR fluorescent images.*

a. Using Fiji2 (ImageJ), load the images of the differentiation wells and the macro entitled “Lipid Droplets.ijm.”

b. In Microsoft Excel, open “FAP_Diff_Analysis.xlsx” and select the *Lipid Droplets* tab.

*Note: All three technical replicates for each condition can be logged on the same sheet; this sheet should be duplicated for each condition to be analyzed.*

c. In Fiji, click *Run* in the macro panel.

d. When prompted, adjust the *brightness and contrast* of the images to reduce the background and then click *Ok* when finished.

e. When prompted, adjust the lipid droplet *threshold* such that all the droplets are included, but any background fluorescence is not. Click *Ok* when finished.

f. When prompted, paste the lipid droplet results into columns A–B of the “Lipid Droplets” sheet. Repeat steps C4.2a–f with all technical replicates, pasting into the subsequent columns.

g. Use the calculated outputs in columns J–K for further analysis.

**D. Myogenic differentiation of MuSCs**

**D1. MuSC culture and differentiation**

1. The following steps should be completed in a biosafety cabinet after cells have been counted according to section B1.

2. Seed 50,000 MuSCs in 100 µL of BoGrow per well in triplicate in a 96-well plate.

a. Optional: Create a negative control well by seeding a fourth well with 50,000 cells in 100 µL of BoGrow in the opposite corner of the same 96-well plate, or on another 96-well plate. Fix these cells according to section B2 after 24 h.

3. Incubate cells at 37 °C and 5% CO_2_ for 24 h.

4. Twenty-four hours post-seeding, carefully aspirate the BoGrow from each well, making sure not to touch the bottom of the wells.

5. Gently add 100 µL of BoDiff media to each of the wells. Return cells to the incubator and let them differentiate for 5 days.

*Note: It is recommended to refresh the BoDiff media on day 3 of differentiation by repeating steps D1.4–5. Differentiation can be visually checked by looking at cell morphology. Differentiated cells should have fused into long tubes (myotubes) and generally appear more elongated. Refer to Figure 3.*

6. After 5 days of differentiation, proceed to fixation as detailed in section B2.

*Note: As MuSCs differentiate, they begin to become contractile and tend to lift off the plate. Adding or removing solutions from wells should be done gently to mitigate loss of myotubes.*

**D2. MuSC staining and imaging**

1. Stain fixed MuSC differentiation plates as detailed in section B3. The following primary and secondary stains should be used:

a. Primary stains: DesB-7 for desmin (1:250 dilution) and MyoG polyclonal for myogenin (1:200 dilution).

b. Secondary stains: donkey anti-mouse 488 antibody (1:500 dilution), donkey anti-rabbit 647 antibody (1:500 dilution), and acti-stain (1:250 dilution).

2. Following staining, follow the steps below for imaging:

a. Hoechst stain for nuclei should be imaged at a wavelength of 461 nm.

b. MyoG should be imaged at a wavelength of 674 nm.

c. Desmin should be imaged at a wavelength of 488 nm.

d. Acti-stain for actin should be imaged at a wavelength of 555 nm.

**D3. Image analysis**

1. Before proceeding, ensure you have the IJM macro “Diff Analysis-MyoG&Des.ijm,” your image files (.lif or .tiff), and the Excel “Diff Template (MyoG&Des).xlsx” downloaded. The macro and Excel template can be found in the supplemental files, and a visual reference for this analysis can be found in Figure 6. Then, follow the directions below:

2. Using Fiji2 (ImageJ), load in the images of the differentiation wells and the macro entitled “Diff Analysis – MyoG&Des.ijm.”

3. In Microsoft Excel, open “Diff Template (MyoG&Des).xlsx.”

4. In Fiji, click *Run* in the macro panel.

5. When prompted, adjust the *brightness and contrast* of the images to reduce the background and then click *Ok* when finished.

6. When prompted, adjust the nuclei *threshold* such that all the nuclei are included, but any background fluorescence is not. Click *Ok* when finished.

7. When prompted, paste the nuclei results into the first 4 columns of the Excel template. Click *Ok* when finished.

8. When prompted, paste the MyoG results into columns H–L in the Excel template. Click *Ok* when finished.

9. When prompted, paste the Desmin results into columns O–S in the Excel sheet. Click *Ok* when finished.

10. Use the *Mean* column in the MyoG section of the Excel sheet and the ROI manager in Fiji to determine the MyoG threshold.

*Note: When determining the threshold, find the point at which the selected stain is just barely visible.*

11. Type the MyoG threshold number into cell J3 of the Excel sheet.

12. Repeat this threshold determination process with Desmin.

13. Use the calculated outputs in columns V–X for further analysis.

## Validation of protocol

This protocol has been used and validated in the following research article:

- Gish et al. [18]. The impact of extracellular matrix proteins on bovine fibro-adipogenic progenitor cell adhesion, proliferation, and differentiation in vitro. *Physiological Reports* (Figures 1, 5, and 6 and Supplemental Figures 2 and 3).
  The FAP adipogenic differentiation protocol has been optimized from the paper listed above to reduce detachment and enhance lipid accumulation. Please see our supplemental material for more information.
  **Figure 5.**
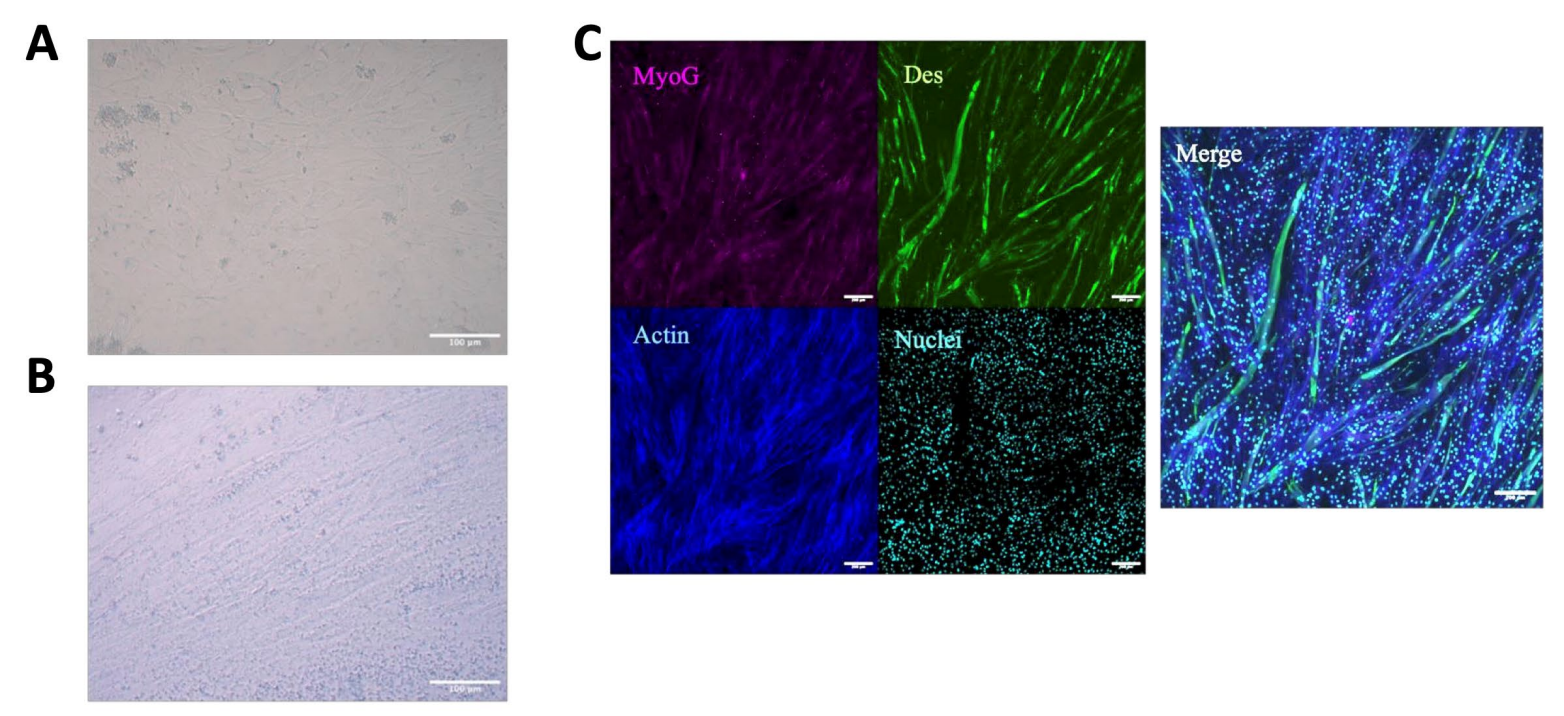
Myogenic differentiation of muscle satellite cells (MuSC). (A) Brightfield image of fixed undifferentiated MuSCs 24 h after seeding. Scale bar, 100 μm. (B) Brightfield image of fixed MuSCs after 5 days of differentiation. Scale bar, 100 μm. (C) Immunofluorescent images of MuSCs after 5 days of differentiation. MyoG in magenta, Desmin in green, actin in blue, and nuclei in cyan. Scale bar, 200 μm.
  **Figure 6.**
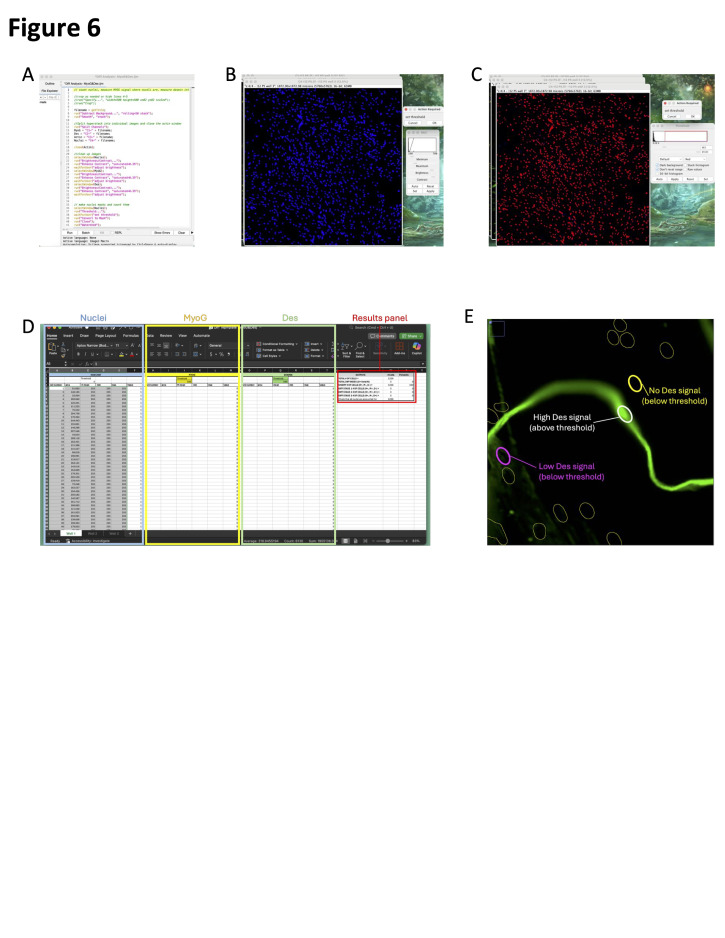
Desmin (Des) and MyoG differentiation assay analysis. (A) DIFF analysis – MyoG&Des.ijm macro upon opening in Fiji. (B) Image of nuclei after brightness/contrast adjustments, showing the module to adjust. (C) Image of nuclei after thresholding, showing the module to adjust. (D) Microsoft Excel template with the areas to paste the nuclei (blue), MyoG (yellow), and Desmin (green) data from Fiji highlighted. The area containing the results is highlighted in red. (E) Zoomed-in image of the Des channel; circles indicate the nuclear mask. The highlighted yellow circle represents a nucleus not within a myotube, the magenta circle represents a nucleus expressing low Des signal, and the white circle represents a nucleus within a myotube expressing high levels of Des.

## General notes and troubleshooting

**General notes**

1. FBS is variable, and a single lot number should be used for all media within the same experiment.

2. Potential sources of variation when using primary bovine cells include, but are not limited to, animal variation (sex, breeding status, breed, age at slaughter, individual) and muscle type/sampling site location. For cell culture experiments using primary bovine MuSCs or FAPs, the use of multiple biological replicates from similar cows and the same sampling muscle/location is imperative to reduce variation and ensure data is applicable to bovine muscle resident stem cells in general.

3. The time between tissue collection and isolation commencement will affect the viability of cells. Prompt initiation of the isolation procedure is recommended for the best results.

4. Fatty acid extracts solubilized in BSA or ethanol may be added to ADM and MM to enhance lipid accumulation. The authors can only endorse concentrations up to 150 mM without fear of lipid toxicity. It is important to note that enhanced lipid accumulation could be due to an uptake of exogenous fatty acids rather than other mechanisms.

5. Bovine MuSCs and FAPs have also been successfully cultured on TC plastic in our lab, though we advise the use of fibronectin. Fibronectin decreases cell attachment time from this isolation to as quickly as 3 h, while collagen or plastic coating can take 3–5 days for multiple cells to become adherent. Please see [18] for more information on culture coatings and bovine FAPs.

**Troubleshooting**

**Problem 1:** Sample foams/bubbles in the digestion phase and/or leaks out of the MACS C tube.

Possible cause: Collagenase or DMEM was not properly warmed.

Solution: The digestion will proceed fine, but some extra cleanup may be required, and the pressure must be relieved before each spin cycle. With a Kimwipe over the lid of the MACS C tube, slowly twist to release any pressure and clean up any ejected liquid. Take care not to fully open the MACS C tube and simply clean the bottom rim where the lid meets the tube. Once cleaned, close the tube and proceed.

**Problem 2:** Isolated cells are found floating in media and not attaching within days 0–3.

Possible causes: Longer isolation duration/rough isolation has added extra stress to the cells. Too much debris remains, interfering with cell attachment.

Solution: Transfer supernatant to a new vessel and add 50% fresh media. To the previous vessel, add fresh media but do not wash to avoid detaching any newly adhered cells. The supernatant plate may continue up to 3 additional days, and any adhered cells can be combined with the original plate at passaging if they have the same passage number.

**Problem 3:** Large amount of debris.

Possible cause: Incomplete debris removal during isolation.

Solution: Once cells are adherent, add an extra 2–3 washes with PBS at each media change or as needed. Hand-aspirate any old media/washes during P0. Debris will be diminished during washes and passaging of cells, and should be nominal at P3, even with incomplete debris removal during isolation.

**Problem 4:** Contamination in freshly isolated cells.

Possible causes: Improper sterile technique, contaminated reagents/incubator, improper ethanol sterilization of tissue.

Solution: If contamination is rampant, dispose of the cells and destroy all reagents used to avoid spreading contamination. If contamination is caught early, removal may be attempted, but it is not always successful. Isolate all reagents used and any contaminated plates, and filter-sterilize all media. Clean and disinfect the incubator and biosafety cabinet. In a 96-well plate, add 100 μL of BoGrow to 15 wells and 10 μL of contaminated media to each well. To individual wells, add increasing volumes of each antibiotic/antimycotic (e.g., 5% Pen/Strep, 20% Pen/Strep, etc.), return to the incubator, and observe contamination growth the following day to assess which is most effective. Wash each affected plate with PBS antibiotic for ~5 min daily. If contamination is adherent, all affected plates should be passaged immediately to fresh plates. If contamination responded to an enhanced antibiotic/antimycotic concentration in the 96-well test, you may add the enhanced amount on top of the regular BoGrow formulation. Saving the cells via passaging and adding antibiotics/antimycotics should not be attempted past 1 week and should only be tried when contamination is minimal or cells are irreplaceable. Proceed with extreme caution; added concentrations of antibiotics/antimycotics may also have cytotoxic effects.

## Supplementary information

The following supporting information can be downloaded here:

1. Figure S1. Bovine FAP adipogenic protocol testing

2. Figure S2. FAP lipid accumulation under varying insulin concentrations

3. Diff Template (MyoG&Des).xlsx

4. Diff Analysis – MyoG&Des.ijm

5. FAP_Diff_Analysis.xlsx

6. bofap_totalarea_diff.ijm

7. Lipid Droplets.ijm
